# Clinician-related factors behind the decision to extract an asymptomatic lower third molar. A cross-sectional study based on Spanish and Portuguese dentists

**DOI:** 10.4317/medoral.21634

**Published:** 2017-08-16

**Authors:** Daniela Alves-Pereira, David Pereira-Silva, Rui Figueiredo, Cosme Gay-Escoda, Eduard Valmaseda-Castellón

**Affiliations:** 1 DDS, MS, Master of Oral Surgery and Implantology. Assistent Professor of Oral Surgery, School of Medicine, University of Coimbra, Portugal; 2 DDS, MS, Master of Orthodontics. Clinical practice in Orthodontics, Coimbra, Portugal; 3 DDS, MS, PhD, Master of Oral Surgery and Implantology. Associate Lecturer in Oral Surgery, Lecturer on the Master of Oral Surgery and Implantology degree course. School of Dentistry, University of Barcelona. Researcher at the Idibell Institute. Barcelona, Spain; 4 MD, DDS, MS, EBOS, OMFS, PhD, Chairman and Professor of Oral and Maxillofacial Surgery, School of Dentistry, University of Barcelona. Director of Master’s Degree Program in Oral Surgery and Implantology (EHFRE International University/FUCSO). Coordinator/Researcher of the Bellvitge Biomedical Research Institute (Idibell). Head of Oral Surgery, Implantology and Maxillofacial Surgery; 5 DDS, MS, PhD, EBOS. Tenured lecturer in Oral Surgery, Director of the Master of Oral Surgery and Implantology. School of Dentistry degree course, University of Barcelona. Researcher at the Idibell Institute. Barcelona, Spain

## Abstract

**Background:**

Scientific literature estimates that around 18 to 40 % of asymptomatic third molars are extracted. The aims of the present study were to determine the indications for extraction of asymptomatic lower third molars in a sample of Spanish and Portuguese dentists, and to relate these indications to the clinicians’ training and professional experience.

**Material and Methods:**

A survey consisting of 15 cases of asymptomatic lower third molars was emailed to Portuguese and Spanish dentists. The clinicians were asked to assess the level of difficulty of the extractions and to make a reasoned recommendation based on the panoramic radiographs, gender and age of the patients.

**Results:**

381 clinicians filled in the questionnaires. Most of the professionals had over 13 years of clinical experience. The number of Spanish clinicians with postgraduate degrees in Oral Surgery was significantly higher. On average, 42% of respondents recommended extraction of asymptomatic third molars. The indication for extraction was significantly higher among Portuguese dentists. Clinical experience was negatively correlated with the perceived extraction difficulty (*p*<0.05). The main reason given for extracting was the prevention of pericoronitis, whereas that for not extracting was the absence of a clear indication and the risk of injuring the inferior alveolar nerve.

**Conclusions:**

The Portuguese dentists were more in favour of removing asymptomatic lower third molars than the Spanish dentists, although the latter had a higher proportion of professionals with postgraduate studies in Oral Surgery.

** Key words:**Third molar, Tooth extraction, Oral Surgery, Indication, Prophylactic removal.

## Introduction

Approximately two-thirds of the world population has at least one third molar (3M) by the age of 20 years (1). In Europe, this is the case in approximately 73% of subjects (2). Extraction of these teeth is the most common surgical procedure in dentistry: it is estimated that around 1 million extractions are performed annually in the United Kingdom and 5 million in the USA (3). In the year 2000, the clinical guidelines of the National Institute for Health and Care Excellence (NICE) in the United Kingdom recommended specific indications for this surgery, at a time when prophylactic extraction was considered unadvisable (5)

However, on looking carefully at the impact of these practice guidelines, although 3M extractions decreased in the years following their introduction the current figures are again similar to those existing before the guidelines were published (4,5). Furthermore, in 2007 the American Association of Oral and Maxillofacial Surgeons (AAOMS) revised the core clinical aspects relating to 3Ms and their extraction. The working group of the AAOMS estimated that 85% of asymptomatic 3Ms were extracted in later stages. Such fragmented criteria result in dentists making different decisions as to whether or not to extract an asymptomatic 3M, probably due to different risk and benefit assessments.

Reports in the scientific literature indicate that around 18% to 40% of 3Ms are extracted without any pathological sign. The decision is based on the need to minimise the future risk of tooth pathology and to reduce age-related surgical morbidity (6). Although some publications compare extraction indications between clinicians, there is very little information about the factors (related either to the patient or the dentist) that influence the decision to extract an asymptomatic 3M (6-11).

The objectives of the present study were to look into the indications for extracting asymptomatic lower 3Ms in a sample of Spanish and Portuguese dentists, using a survey, and to link such indications to the clinicians’ training and expertise.

## Material and Methods

A request to participate in a survey consisting of 15 clinical cases was sent by email to dentists in Spain and Portugal. The questionnaire was available at a survey server (http://www.surveygizmo.com) and could be filled on-line.

In Spain, the request was made through professional associations of dentists in the different regions of the country. In Portugal, it was managed through Box4®, a private company dedicated to medical and dental information, which has a mailing list of all the dentists in the country. The questionnaire was first tested by a group of 6 dentists to ensure that the information in the survey was accurate, there were no misunderstandings and the results were recorded correctly. The surveys were completed during a 6-month period, between July 4, 2013 and January 23, 2014. This study was not submitted to an Ethics Committee since it was based on an anonymous survey made to dentists and the patients’ used in this study were fictitious.

First, each dentist had to fill in personal, demographic and professional information. A clinical summary of 15 clinical cases of young healthy patients with 29 asymptomatic 3Ms was then shown, including panoramic radiographs and the age and gender of the patient. The cases were presented in random order to each respondent. The 3Ms represented a broad spectrum of asymptomatic third molars ( Table 1). The respondent was asked to make a recommendation for each case (whether or not to extract) and to select a reason for that decision from a closed list of options. The clinicians also had to rate the extraction difficulty of each lower 3M on a Likert scale of 1-10.

**Table 1:**
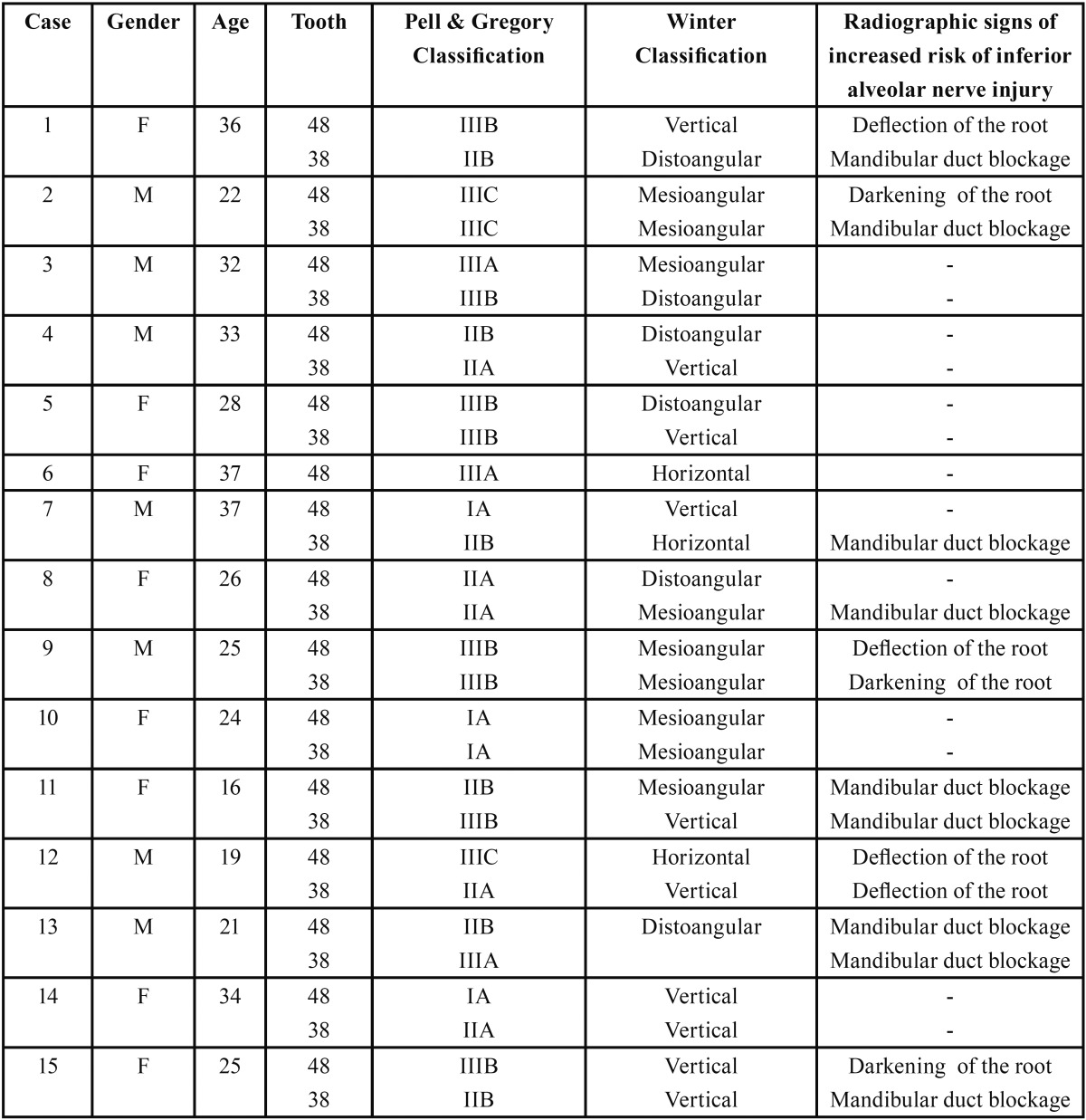
Description of age, gender (M: Male; F: Female) and radiographical landmarks (position and inclination of 3M according to the Winter and Pell and Gregory classifications, degree of soft tissue coverage and relationship with the mandibular canal).

The results were exported to a Microsoft Office® XML® spreadsheet (Microsoft Corp., Redmond, Washington, USA), accidentally duplicated replies were eliminated and the results were analysed using IBM SPSS® 22.0 software (IBM Corp., New York, USA). Chi-square tests (categorical variables), Student’s t-tests and Pearson’s correlation for scale variables were used. The level of significance was set at *p*<0.05.

## Results

Three hundred and ninety-six (396) clinicians completed the online questionnaire. The Spanish and Portuguese dentists were divided into two subgroups according to their country of residence (regardless of their nationality). There were 230 dentists working in Portugal, 151 in Spain and 15 in other countries. The latter were discarded because they were considered a small and heterogeneous group. Of the 381 respondents included in the study, 209 were female and 172 male.

Most of the dentists had over 13 years of clinical experience. The median graduation year was similar in the Spanish and Portuguese dentists (2003).

In this sample, 12.4% of the respondents had a teaching position at a University. This figure was significantly higher (*p* = 0.045) among Portuguese dentists (14.8% vs. 7.9%).

**Figure 1:**
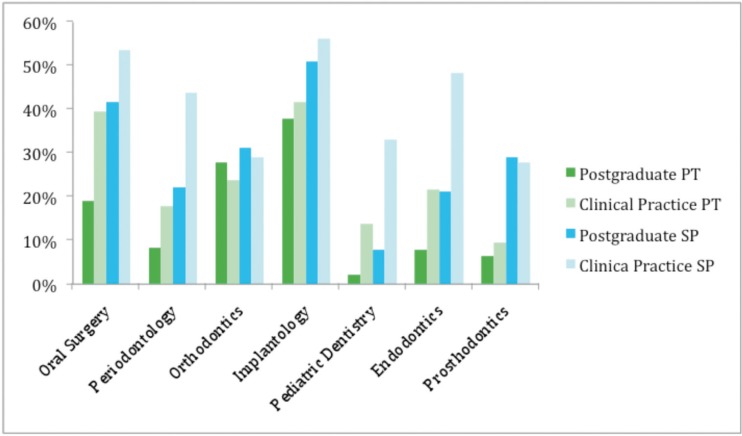
Distribution of the Portuguese (PT) and Spanish (SP) respondents according to their daily clinical practice and postgraduate education.

Most (74.8%) were general practitioners but many had received training in some specific field. Indeed, 45.1% of the general dentists practised Oral Surgery, 28.1% Periodontology and 26% Orthodontics. The proportion of Orthodontics postgraduates was 29.1%, clinicians with a postgraduate degree in Oral Surgery were 28.1% and Periodontics postgraduates were 13.4% of the sample (Fig. 1).

In Spain, the proportion of dentists with a postgraduate diploma in Oral Surgery who completed the survey was significantly higher than in Portugal (41 .7% vs. 19.1%. *p* = 2 -10-6), which reflects that in Spain more respondents practised Oral Surgery (53.6% vs. 39.6%) (*p* = 0.007).

The estimated difficulty of 3M surgery was negatively correlated with experience: the most recent graduates rated the cases as more difficult. On the other hand, Portuguese dentists found lower 3Ms easier to extract than their Spanish colleagues (*p*<0.05).

On average, the dentists recommended extraction of 42% of the asymptomatic third 3Ms (38.3% in Spain and 44.5% in Portugal). Fewer Spanish than Portuguese clinicians recommended extractions in 23 of the 29 3Ms, although the difference was statistically significant in only 8 out of the 29 3Ms (*p*<0.05).

None of the clinicians considered the probability of cyst formation as an indicator for asymptomatic lower 3M removal. In contrast, the risk of pericoronitis was the main reason provided for lower 3M extraction ( Table 2).

**Table 2:**
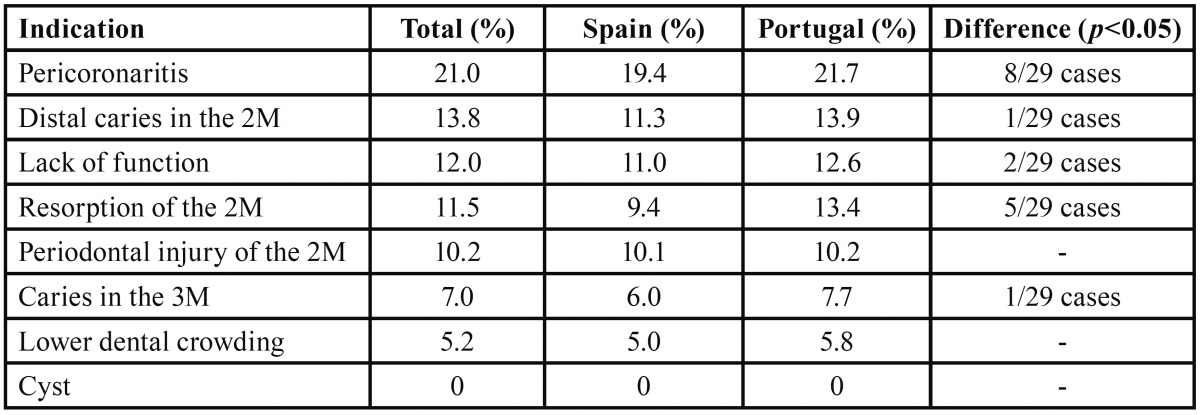
Core indications mentioned by the Portuguese and the Spanish clinicians for extracting asymptomatic lower 3Ms.

More than 30% of all the clinicians found extraction unnecessary because they did not foresee complications in the short to medium term. After this, the most frequent reason for not recommending the removal of an asymptomatic 3M was the tooth’s proximity to the lower alveolar nerve and the risk of nerve injury. Spanish clinicians expressed this concern more often than Portuguese dentists ( Table 3).

**Table 3:**
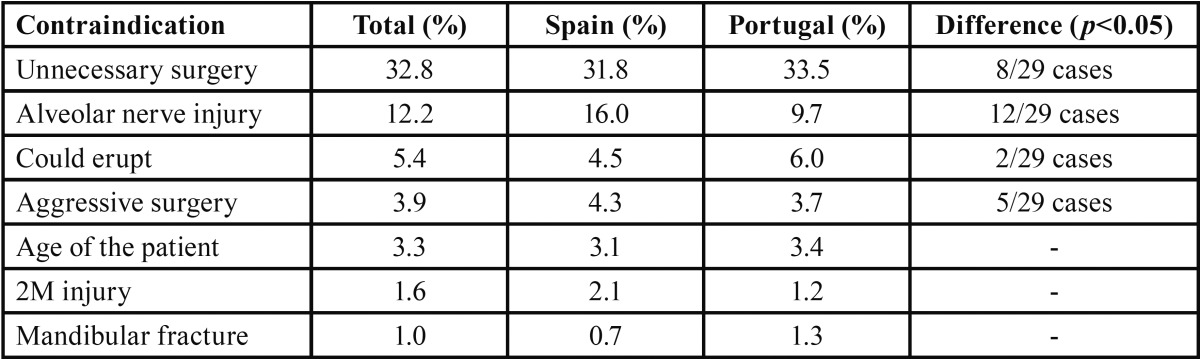
Core reasons mentioned by the Portuguese and Spanish clinicians for not extracting asymptomatic lower 3Ms.

When the decision not to extract a third molar was taken, patient age was the reason least often given by both Spanish and Portuguese clinicians, except in one case of a 16-year old patient with two germs. In this case, 18% of respondents mentioned age as a reason for not recommending extraction. Of all the Portuguese respondents, 20% assumed that there was apparently no need to extract tooth germs and around 30% stated that these teeth could still erupt (*p* <0.05). About 16% of Spanish respondents believed that the tooth germs could still erupt.

## Discussion

Although there appears to be a positive correlation between the decision to remove a third molar and the risk of developing a disease (6), the dentist’s ability to predict such a risk is very limited. The present study showed that Portuguese clinicians tend more towards extraction. This may be because Portuguese dentists considered the 29 3M extraction simpler than did their Spanish colleagues. There appears to be a correlation between the 3M extraction difficulty, its position in relation to the ascending ramus of the mandible (2) and patient age (11). Other factors like the surgeon’s experience also play a crucial role. Therefore, clinicians are advised to have excellent technical and anatomical skills before performing surgical procedures (12). Knutsson *et al.* (9) highlighted that general dental practitioners — who comprised the vast majority of our sample (74.8%) — had limited access to scientific papers, while postgraduates were apparently more familiar with both international literature and the analytical tools needed to interpret and analyse outcomes. In the sample from Portugal, a higher proportion of professionals with teaching activities replied to the survey, but most of them were not experts in Oral Surgery. In contrast, Berrocal *et al.* (13) analysed students’ perceptions of the Oral Surgery curricula taught in Spanish public Universities and concluded that most undergraduates found it to be adequate, except for the most complex surgical procedures. To our knowledge, there are no similar courses in Portugal.

In this study, the proportion of dentists with oral surgery postgraduate degrees was much higher in Spain. This marked difference is not due to a bias in survey distribution, but to general dentists apparently not responding to the questionnaire to the same extent as in Portugal. It must also be mentioned that in Spain and Portugal at the time of the survey there was no official specialisation in oral surgery, and the content of the respondents’ postgraduate education could be very different. The imbalance between dentists with and without postgraduate diplomas in oral surgery could explain the observed differences between Spanish and Portuguese dentists better than the country in which they practiced (10). The more conservative attitude might be due to a better knowledge of the practice guidelines that do not recommend prophylactic removal of 3M. In addition, practising oral surgery on a daily basis seems to help maintain surgical skills (12). These variations between clinicians from two different countries had already been seen in another comparative study involving the UK and Hong Kong (8).

The present study also proved that experience enhanced confidence (the difficulty was rated lower), probably due to increased surgical skills arising from years of professional activity (14).

Although the clinicians in this study did not recommend third molar removal to prevent cyst formation, the scientific literature shows that the prevalence of cyst and tumour development around lower 3Ms ranges between 2% and 6.2% in the long term (15). Although the risk factors for cystic or tumour development are unknown, pericoronal radiolucencies wider than 2.5mm seem to dysregulate cell death and increase anti-apoptotic bcl-2 protein activity (16), which increases the likelihood of pathological changes arising in the follicle. In this case, extraction could be considered. On the other hand, radiographies do not seem to be an appropriate tool for diagnosing pathological changes and biopsy is recommended (17).

Pericoronitis was indicated as a potential complication by 21% of the clinicians. However, many indications concerned fully impacted molars, which are not at risk of pericoronaritis unless it is assumed that eruption can still occur. In fact, few 3Ms remain static and their position changes over time, although this does not necessarily imply eruption (18).

There are still clinicians who support 3M extraction to prevent late anterior-inferior crowding (19). However, most of the studies published in the last few years have failed to find any association between the eruption of 3M and crowding of the anterior teeth (20).

The current literature suggests a low prevalence of second molar (2M) external resorption (0.3 to 7%), although this percentage can be 4 times higher if, instead of analysing panoramic radiographies, Cone Beam Computed Tomographies (CBCT) are used (21). In the present study, 2M resorption was selected by more than 11% of the clinicians as an indication for removing the 3Ms.

The percentage of 3Ms extracted due to caries on the distal side of 2M in young patients seems to be very low (2-5%). However, in older patients the proportion increases to up to 30% (22). In the present study, 13% of the respondents though that the risk of caries in the 2M was an indication for extraction. Almost 2/3 of professionals cited 3Ms in a horizontal position and classified as Pell & Gregory IIIA as high-risk cases. Indeed, the probability of developing caries in the distal aspect of the 2M increases when the angulation between the 3M and the 2M is between 43º and 71º (23), or if the distance between the cement-enamel junction of the two teeth is between 3 and 10 mm (24). Several authors have also stressed the importance of age in the development of this complication (27). In fact, according to Kang *et al.* (23) patients older than 27 have double the risk of caries in the second molar. This reinforces the idea that poorly positioned 3Ms carry a higher caries risk and that it increases with age because of a cumulative effect (24).

Regarding the periodontal health of 2Ms, 10.2% of clinicians believed that 3M extraction was beneficial, against 1.6% who found that surgery would only make it worse. Both opinions are supported by clinical evidence (25).

In the present study, 57.9% of clinicians chose not to remove asymptomatic 3Ms. The main reason was that extraction did not seem necessary, the tooth was still functional or no complications were foreseeable in the short or medium term. The risk of inferior alveolar nerve injury was the second reason given against extraction. Although the literature shows a low incidence of nerve injuries, ranging from 0.4% to 8%, 25% of cases with nerve injury have irreversible symptoms (26). The Spanish clinicians were more concerned about this complication, probably because they were more familiar with these injuries and the associated risk factors (9).

The risk of mandible fracture and the fact that surgery was too aggressive were also referred to as contraindications for the extraction of 3Ms. However, studies show that fracture is an extremely rare complication and is more common in older patients with fully impacted 3Ms in specific positions (II-III C) (27).

Notwithstanding the methodological constraints of this study, it may be concluded that years of professional experience were negatively correlated with the perceived difficulty of 3M extraction. In general, surprisingly, dentists in both countries did not have a conservative attitude, but Spanish dentists, who had more oral surgery training, recommend fewer extractions of asymptomatic 3Ms. The risk of pericoronaritis and distal caries in the 2M were the main reasons for an indication to extract, while the risk of alveolar nerve injury and the aggressiveness of the surgery were the main contraindications.
